# Rationally introduce multi-competitive binding interactions in supramolecular gels: a simple and efficient approach to develop multi-analyte sensor array†
†Electronic supplementary information (ESI) available: Experimental details and characterization data; gelation properties; fluorescence spectra of the supramolecular gels and the supramolecular gel-based sensor array response for guest ions; fluorescence titrations for target ions. See DOI: 10.1039/c6sc00955g


**DOI:** 10.1039/c6sc00955g

**Published:** 2016-04-25

**Authors:** Qi Lin, Tao-Tao Lu, Xin Zhu, Tai-Bao Wei, Hui Li, You-Ming Zhang

**Affiliations:** a Key Laboratory of Eco-Environment-Related Polymer Materials , Ministry of Education of China , Key Laboratory of Polymer Materials of Gansu Province , College of Chemistry and Chemical Engineering , Northwest Normal University , Lanzhou , 730070 , China . Email: linqi2004@126.com ; Email: zhangnwnu@125.com

## Abstract

Sensor arrays are a powerful tool for multianalyte sensing and the development of an efficient sensor array has become one of the most intriguing problems. However, sensor arrays often employ lots of receptors which need large amounts of work to synthesise. This study describes an efficient method for the fabrication of a simple sensor array based on the competitive binding in supramolecular gels. By rationally introducing various well-designed competitive binding interactions into the supramolecular gel, which is self-assembled from a naphthylhydrazone-based organogelator, a supramolecular gel-based twenty-two-member sensor array has been created. Interestingly, the sensor array has been shown to accurately identify fourteen kinds of important ions (F^–^, Cl^–^, I^–^, CN^–^, HSO_4_^–^, SCN^–^, S^2–^, OH^–^, Al^3+^, Fe^3+^, Zn^2+^, Hg^2+^, Pb^2+^ and H^+^) in water. It's important to note that this sensor array needs only one synthesized receptor. Moreover, using this method, we also obtained a series of ion response fluorescent supramolecular materials, which could act as security display materials. Therefore, it's a novel and facile way for the design of a simple sensor array as well as ion response fluorescent supramolecular materials.

## Abstract 2

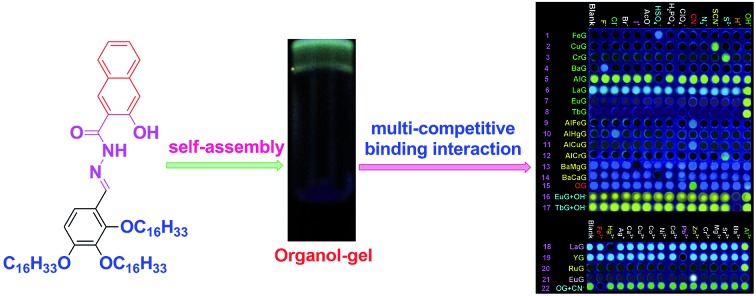
A supramolecular-gel-based twenty-two-member sensor array has been created by introducing well-designed multi-competitive binding interactions into a supramolecular gel.

## Introduction

In the last decade, interest in molecular sensing has been slowly shifting from selective sensors toward sensor array-based multianalyte sensing because lots of the sensor arrays have been shown to be highly efficient in various analyte detection.^1^ In general, a sensor array is based on a series of sensors which could recognize a number of analytes with a high classification accuracy, there are several wonderful reviews presenting the development of sensor arrays.1a–f Up to now, lots of strategies have been developed for the creation of sensor arrays. For instance, Anzenbacher *et al.* developed a series of sensor arrays for the detection of anions,^2^ and carboxylate drugs.^3^ Suslick *et al.* created a series of sensor arrays for the detection of organic compounds,^4^ volatile organic compounds (VOCs)^5^ and nanoparticles.^6^ Usually, a high-performance sensor array employs lots of individual selective receptors which need large amounts of work to design, synthesise and pre-research their sensing properties.1a–c In order to overcome the difficulties associated with the preparation of various individual receptors, we need to develop a novel strategy to design simple but highly efficient sensor arrays. Could we develop a sensor array which is based on only a single synthesized receptor? It's a really interesting task.

Moreover, ions play a fundamental role in chemical, biological and environmental processes,^7^ however, reliable sensing of different ions with a similar structure in water is a difficult problem.^2^,^7^ Although there are numerous chemosensors reported every year the development of an efficient sensor array recognizing a number of ions with a high classification accuracy is still a intriguing challenge.

Fortunately, the rapid development of stimuli-responsive supramolecular gels^8^–^11^ provides a novel platform for the design of efficient sensor arrays. As we all know, on account of the dynamic and reversible nature of self-assembly in supramolecular gels, stimuli–responsive supramolecular gels can sense, process, and actuate a response to an external change without assistance.^8^–^11^ These excellent properties make supramolecular gels a wonderful candidate for chemosensors. However, although many supramolecular gel-based chemosensors have been reported, reports on supramolecular gel-based sensor-arrays are very rare.1a–f,k More interestingly, in our previous work, we found that the stimuli–responsive properties of the supramolecular gels could be efficiently controlled by the competitive coordination of the gelator with the metal ions.^11^ These fine properties provide the supramolecular gel with good opportunities to act as sensor arrays. In view of these, in this study, by rationally introducing various well-designed competitive binding interactions into a functionalized organogel, we created a novel supramolecular gel-based twenty-two-member sensor array. The sensor array could accurately identify fourteen kinds of ions in water, ions including F^–^, Cl^–^, I^–^, CN^–^, HSO_4_^–^, SCN^–^, S^2–^, OH^–^, Al^3+^, Fe^3+^, Zn^2+^, Hg^2+^, Pb^2+^ and H^+^. It is worth mentioning that the sensor array was constructed using only one receptor. Simply stated, in the twenty-two-member sensor array, the organogelator not only acts as a gelator, but also as a receptor. The selective sensing properties of the sensor array are accurately controlled by the various competitive binding interactions which we rationally induced in the gel.

## Results and discussion

**Fig. 1:**
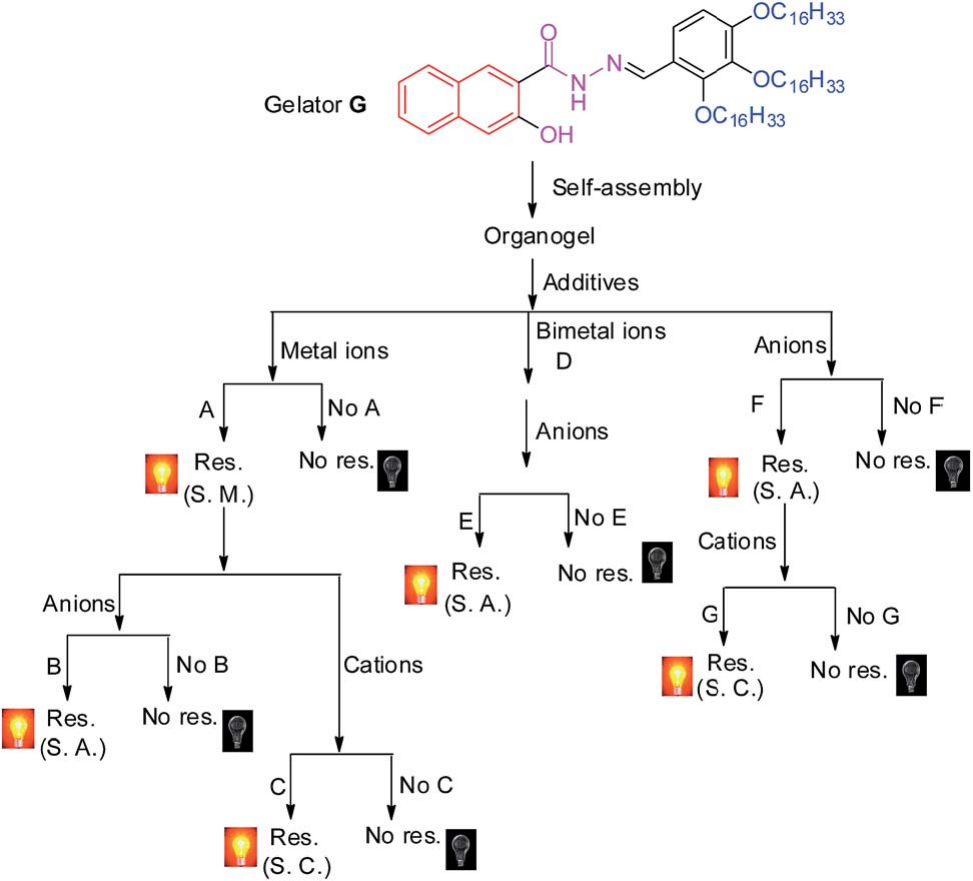
Chemical structure of **G** and the competitive binding interactions: (A) metal ions competitively coordinate with the gelator; (B) anions competitively coordinate with the metal ions or gelator; (C) competitive coordination between different metal ions and the gelator; (D) bimetal ions competitively coordinate with the gelator; (E) anions competitively coordinate with the bimetal ions; (F) anions competitively binding with the gelator; (G) cations competitively coordinate with the anions or gelators. Note for abbreviations: Response (Res. or res.), Sensing for anions (S. A.), Sensing for Cations (S. C.) and Sensing for Metal ions (S. M.).

Firstly, as show in Fig. 1, we rationally designed and synthesized a multi-functionalized organogelator **G** by introducing a coordination site, multi-self-assembly driving forces, and fluorescent signal groups into the gelator molecule. For example, we introduced an acylhydrazone group and a hydroxy group as the coordination, hydrogen-bonding and recognition sites, a naphthyl group as the signal group and π–π stacking site and long alkyl chains as the strong van der Waals forces group. The gelation abilities of **G** were examined in various solvents by means of the “stable to inversion of a test tube” method (Table S1†). As we expected, **G** showed excellent gelation abilities in various solvents such as dimethyl formamide (DMF), dimethyl sulfoxide (DMSO), acetone, ethanol, *n*-propyl alcohol, isopropanol, *n*-butyl alcohol, isoamyl alcohol, *n*-hexanol, cyclohexanol, ethyl acetate, CCl_4_, petroleum ether and so on. Among these solvents, the gelator **G** showed the lowest critical gelation concentration (CGC) (0.2%, w/v, 10 mg ml^–1^ = 1%) and the highest gel–sol transition temperature (*T*_gel_) in *n*-butyl alcohol (Table S1†). Therefore, the **G** organogel formed in *n*-butyl alcohol is more stable than the gels in other solutions and we carried out subsequent studies on the **G** organogel (named **OG**) formed in *n*-butyl alcohol.

**Fig. 2:**
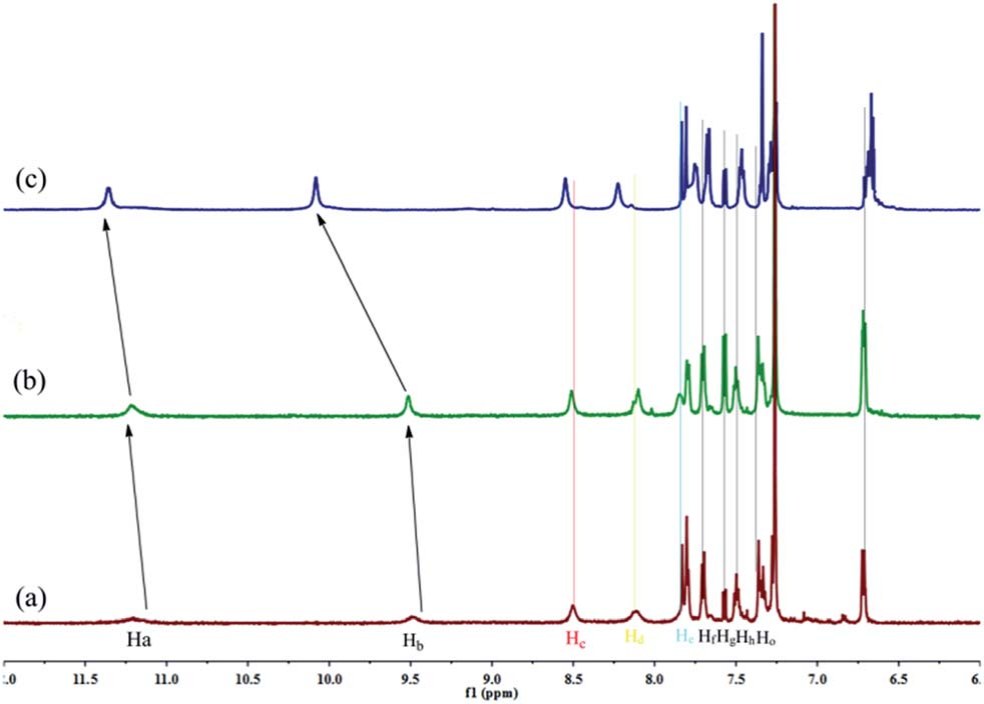
Partial ^1^H NMR spectra of **G** in CDCl_3_ with different concentrations, (a) 10 mg ml^–1^; (b) 20 mg ml^–1^; (c) 30 mg ml^–1^.

The self-assembly mechanism of **OG** was investigated using ^1^H NMR, IR, X-ray and SEM. In the concentration dependent ^1^H NMR (Fig. 2) of **G**, the –OH (H_a_), –NH (H_b_) and –N=CH (H_c_) resonance signals showed obvious downfield shifts as the concentration of **G** rose. These results revealed that in the gelation process, these groups formed hydrogen bonds with the –C=O and –N=C groups on the adjacent gelators. Moreover, the IR studies also confirmed this result, as shown in Fig. S4a,† in the powder **G**, the stretching vibration of –OH and –NH appeared as a broad peak at 3202 cm^–1^, while, owing to the formation of hydrogen bonds, in the organogel **OG**, these absorptions were shifted to 3380 and 3244 cm^–1^, respectively. On the other hand, with the gradual increase in concentration, the ^1^H NMR signal of the naphthyl protons (H_d_, H_e_, H_f_, H_g_, H_h_ and H_o_) showed an obvious upfield shift, indicating that the π–π stacking interactions between the naphthyl groups were involved in the gelation process.^12^ The X-ray peak (Fig. S5†) at 2*θ* 24.5° (*d* = 3.96) also confirms the π–π stacking interactions. Thus, the gelator **G** self-assembled to organogel **OG** through the hydrogen bonds and π–π stacking as well as the vdW existing in the long alkyl chains. The morphological features of the organogel **OG** were studied using SEM (Fig. S6a†) with its xerogels and showed a rugate layer structure.

**Fig. 3:**
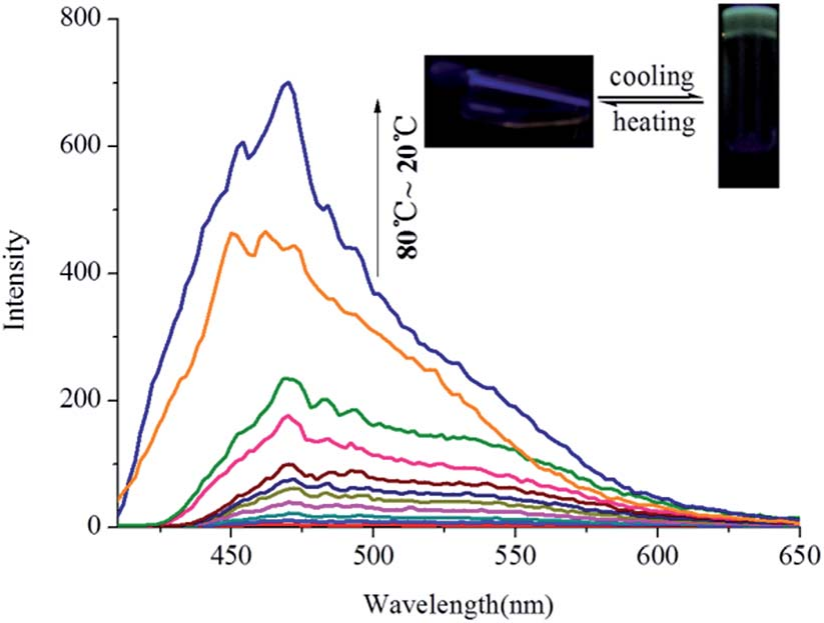
Temperature-dependent fluorescence spectra of **OG** (in *n*-butyl alcohol, 0.8%) during the gelation process (*λ*_ex_ = 300 nm).

Interestingly, **OG** shows aggregation-induced fluorescence emission (AIE)^13^ in the organogel state in various solvents. For example, as shown in Fig. 3, **G** has no fluorescence in hot *n*-butyl alcohol solution (*T* > *T*_gel_). However, with the temperature of hot *n*-butyl alcohol solution dropping below the *T*_gel_ of **OG**, the emission intensity at 475 nm showed a sudden increase and reached a steady state, which indicated that the turquoise fluorescence of **OG** was AIE. Meanwhile, this emission shows a large Stokes shift *ca.* 175 nm (Fig. S7†).

**Fig. 4:**
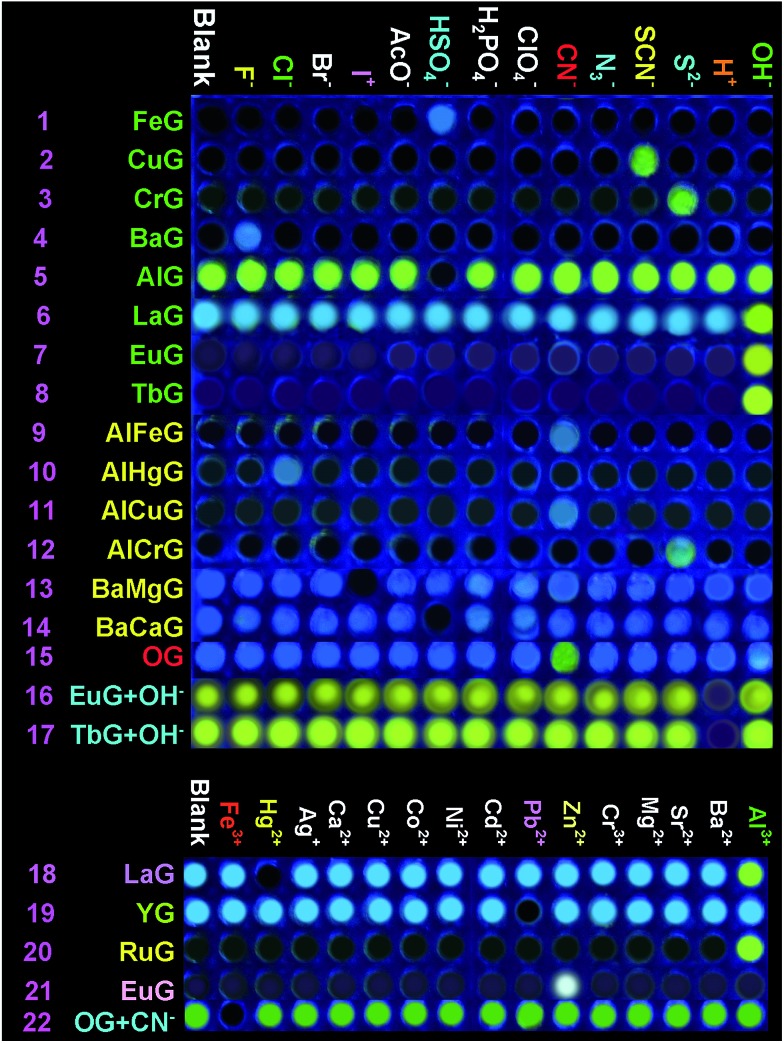
Fluorescence responses of the supramolecular gel-based sensor array to the presence of various anions and cations.

Secondly, in order to develop an **OG**-based sensor array, as shown in Fig. 1, we rationally induced seven kinds of well-designed competitive binding interactions ((A–G) in Fig. 1) into organogel **OG** to control the ion response capabilities of the gel. These competitive binding interactions were introduced by adding various metal ions, bimetal ions, anions or alternatively adding metal ions and anions into **OG** according to Fig. 1, respectively. Interestingly, *via* the gelator and ions taking place through competitive coordination or binding, the supramolecular gel could show a selective response for the target ions (Fig. 4).

At the beginning, we introduced competitive binding interactions between the metal ions and gelators (competitive binding interaction (A) in Fig. 1) by adding various metal ions into **OG**. The addition and diffusion of various metal ions (Mg^2+^, Ca^2+^, Cr^3+^, Fe^3+^, Co^2+^, Ni^2+^, Cu^2+^, Zn^2+^, Ag^+^, Cd^2+^, Hg^2+^, Pb^2+^, Ba^2+^, Sr^2+^, Al^3+^, La^3+^, Y^3+^, Ru^3+^, Eu^3+^ and Tb^3+^) to **OG** generated the corresponding metallogels (named **MGs**, for example **MgG**, **CaG**, **CrG** and so on) respectively (Fig. S8†). Interestingly, as shown in Fig. S8 and S9,† upon the addition of 0.5 equiv. of Cu^2+^, Ba^2+^, Fe^3+^, Cr^3+^, Ru^3+^, Eu^3+^ or Tb^3+^ to **OG**, the AIE of **OG** was quenched and a corresponding no fluorescence metallogel formed; while the addition of 0.5 equiv. of Ca^2+^, Al^3+^, La^3+^, Y^3+^ and so on could induce the AIE of **OG** taking place with obvious shifts. For instance, the metallogel **AlG** emitted a strong green fluorescence, while, **LaG** and **YG** emitted a strong blue fluorescence.

**Fig. 5:**
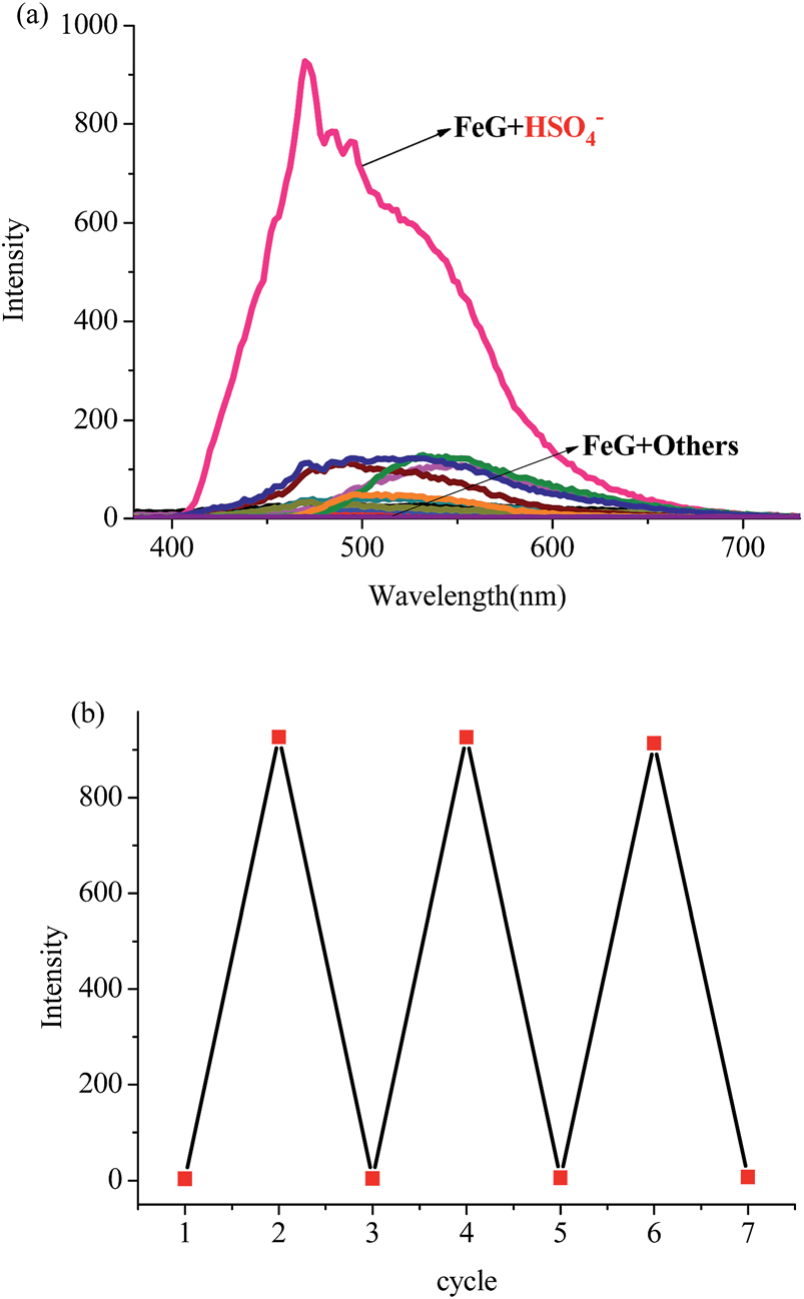
(a) Fluorescence spectra of **FeG** (in the gelated state) in the presence of various anions (5 equiv. of F^–^, Cl^–^, Br^–^, I^–^, AcO^–^, H_2_PO_4_^–^, N_3_^–^, CN^–^, SCN^–^, ClO_4_^–^, S^2–^ and OH^–^). (b) Fluorescence “OFF–ON–OFF” cycles of **FeG** (in the gelated state), controlled by the alternative addition of Fe^3+^ and HSO_4_^–^, *λ*_ex_ = 300 nm.

Then, we introduced competitive binding interactions between the metal ions, anions and gelators (competitive binding interaction (B) in Fig. 1) by adding various anions into the **OG**-based metallogels. As shown in Fig. 4 (line 1) and Fig. 5a, with the addition of water solutions of various anions (F^–^, Cl^–^, Br^–^, I^–^, AcO^–^, H_2_PO_4_^–^, HSO_4_^–^, N_3_^–^, SCN^–^, S^2–^, ClO_4_^–^, CN^–^, and OH^–^) into the metallogel **FeG**, only HSO_4_^–^ could induce the metallogel **FeG** emitting a turquoise fluorescence at 470 nm immediately. While other anions such as F^–^, Cl^–^, Br^–^, I^–^, AcO^–^, H_2_PO_4_^–^, N_3_^–^, CN^–^, SCN^–^, ClO_4_^–^, S^2–^ and OH^–^ could not induce any significant emission changes. Therefore, **FeG** showed selective fluorescence “turn-on” sensing of HSO_4_^–^ in water. The detection limits of **FeG** for HSO_4_^–^ are 1.0 × 10^–6^ M (Fig. S11a and Table S2†).

Similar tests were applied to the other no fluorescence metallogels such as **CuG**, **CrG**, **BaG**, **EuG**, **TbG** and the fluorescence metallogels such as **AlG**, **LaG** and so on. From the results, **CuG**, **CrG**, **BaG**, **EuG**, and **TbG** could selectively fluorescence “turn-on” sense SCN^–^, S^2–^, F^–^ and OH^–^ (Fig. 4) (line 2, 3, 4, 7, 8; and Fig. S10a–c, f and g†) while **AlG** and **LaG** could selectively sense HSO_4_^–^ and OH^–^ (Fig. 4 (line 5 and 6), Fig. S10d and e†) respectively.

Then we introduced competitive binding interactions between the metal ions and gelators (competitive binding interaction (C) in Fig. 1) by adding water solutions of various metal ions (Mg^2+^, Ca^2+^, Cr^3+^, Fe^3+^, Co^2+^, Ni^2+^, Cu^2+^, Zn^2+^, Ag^+^, Cd^2+^, Hg^2+^, Pb^2+^, Ba^2+^, Sr^2+^, Al^3+^, La^3+^, Y^3+^, Ru^3+^, Eu^3+^ and Tb^3+^) into the metallogels. As a result, **LaG** and **YG** could selectively sense Hg^2+^ and Pb^2+^ (Fig. 4 (line 18 and 19), Fig. S11l and n†) while **RuG** and **EuG** could selectively fluorescence “turn-on” sense Al^3+^ and Zn^2+^ (Fig. 4 (line 20 and 21), Fig. S11m and o†) respectively.

In light of the above results that the addition of metal ions such as Cu^2+^, Ba^2+^, Fe^3+^, Cr^3+^ or Ru^3+^ could quench the AIE of **OG** while the addition of Ca^2+^, Al^3+^, La^3+^, and Y^3+^ could induce the AIE of **OG** taking place with obvious shifts or enhancement, we rationally induced two different kinds of metal ions into **OG** to control the AIE of the gel. Due to the different coordination capabilities of the bimetal ions, there is competitive coordination existing in the gelator and the two kinds of metal ions (competitive binding interaction (D) in Fig. 1), therefore, the fluorescence properties of the gel could be well controlled using this competition. For instance, through the synchronous addition of 0.5 equiv. of Al^3+^ and Fe^3+^ into **OG**, we could obtain the corresponding bimetallogel **AlFeG**. Using a similar method, we could obtain a series of bimetallogels such as **AlHgG**, **AlCuG**, **AlCrG**, **BaMgG**, **BaCaG** and so on. Interestingly, as we expected, the fluorescence properties of the bimetallogel depends on the metal ion which has the stronger coordination capability than the other metal ion. For example, because the coordination capability of Fe^3+^ is stronger than Al^3+^, the fluorescence properties of **AlFeG** are similar to those of **FeG**. Therefore, **AlFeG** is a no fluorescence metallogel. Similarly, **AlHgG**, **AlCuG** and **AlCrG** are no fluorescence metallogels while **BaMgG** and **BaCaG** are fluorescence metallogels.

Then, we introduced competitive binding interactions among the bimetal ions, anions, and gelators (competitive binding interaction (E) in Fig. 1) by adding water solutions of various anions into these bimetallogels. As show in Fig. 4 (line 9–14), Fig. S10h–m,† **AlFeG**, **AlHgG**, **AlCuG** and **AlCrG** could selectively fluorescence “turn-on” sense CN^–^ (by **AlFeG** and **AlCuG**), Cl^–^ (by **AlHgG**) and S^2–^ (by **AlCrG**) respectively; while, **BaMgG** and **BaCaG** could selectively fluorescence “turn-off” sense I^–^ and HSO_4_^–^ respectively.

Moreover, we also introduced competitive binding interactions between the anions and gelators (competitive binding interaction (F) in Fig. 1) by adding the water solutions of various anions into **OG**. As show in Fig. 4 (line 15) and Fig. S10o,† with the addition of water solutions of various anions (F^–^, Cl^–^, Br^–^, I^–^, AcO^–^, H_2_PO_4_^–^, N_3_^–^, SCN^–^, S^2–^, ClO_4_^–^, CN^–^, and OH^–^) into **OG** respectively, only CN^–^ could induce the AIE emission of **OG** to take place a significant red shift from 470 nm to 520 nm immediately. Meanwhile, turquoise fluorescence of **OG** changed to yellow. While other anions such as F^–^, Cl^–^, Br^–^, I^–^, AcO^–^, H_2_PO_4_^–^, N_3_^–^, SCN^–^, ClO_4_^–^, S^2–^ and OH^–^ could not induce any significant emission or color changes. Therefore, **OG** could selectively fluorescence and colorimetric sense CN^–^ in water.

Finally, we introduced competitive binding interactions among the anions, metal ions and gelators (competitive binding interaction (G) in Fig. 1) by adding various metal ions into the organogel **OG + CN**, as a result, as shown in Fig. 4 (line 22) and Fig. S10o,† **OG + CN** can selectively sense Fe^3+^.

The detection limits of this supramolecular gel-based sensor array for the corresponding ions were investigated *via* fluorescence titrations (Fig. S11†). As a result, the sensor array shows a high sensitivity for target ions. For example, the bimetallogel sensor **AlCuG** shows a very low detection limit for CN^–^ (1.0 × 10^–7^ M), which is lower than the WHO guideline of 1.9 × 10^–6^ M.^14^ The other detection limits of the sensor array for the corresponding ions are listed in Table S2.†

We investigated the sensing mechanism of the above mentioned metallogels and bimetallogels *via* IR and SEM. For instance, in the FT-IR of **OG** (Fig. S4b†), the –C=O and –N=CH– vibration absorption peaks appeared at 1641 and 1594 cm^–1^ respectively. While, with the addition of Fe^3+^ into **OG** and the formation of **FeG**, these two peaks shifted to 1647 and 1588 cm^–1^ respectively, which was attributed to the coordination of Fe^3+^ with the gelator *via* the acylhydrazone group of the gelator. As we expected, after the addition of HSO_4_^–^ into metallogel **FeG**, the –C=O and –N=CH– vibration absorption peaks returned to 1641 and 1594 cm^–1^ respectively, which indicated that the HSO_4_^–^ competitively binds with Fe^3+^ and releases the acylhydrazone group of the gelator. This result indicated that the ion sensing mechanism of the above mentioned metallogels and bimetallogels is based on the competitive coordination which took place among the gelators, metal ions or anions in the supramolecular gel system. In the corresponding SEM (Fig. S6†), owing to the competitive binding of the Fe^3+^ with gelator **G**, the micromorphology of the supramolecular gels shows obvious changes.

The further response mechanism of **OG** for CN^–^ was investigated *via*^1^H NMR titration experiments and IR. In the ^1^H NMR (Fig. S12†), the –N=CH protons on **G** appeared at *δ* 9.51 ppm. After adding CN^–^, this signal faded away, while, two new signals appeared at *δ* 3.10 and 5.64 ppm, which were attributed to the formation of the NC–CH– and –NH groups, respectively. Meanwhile, in the FT-IR of **OG** (Fig. S4c†), the –N=CH vibration absorption at 1594 nm^–1^ disappeared and a new –C≡N vibration absorption appeared at 2176 cm^–1^. These results indicated that the CN^–^ was added to the imines group on **OG***via* a nucleophilic addition reaction and formed a new organogelator **OG + CN**. During this process, the emission band of **OG** underwent a red shift, which is attributed to the ICT process.

The competitive binding mechanism was also supported by the reversibility of the sensing process. For example, the metallogel **FeG** could selectively fluorescence “turn-on” sense HSO_4_^–^, while, upon the addition of Fe^3+^ into the HSO_4_^–^-containing **FeG**, the fluorescence of **FeG** was quenched, which was attributed to the Fe^3+^ coordination with the gelator again. These properties make **FeG** act as a HSO_4_^–^ and Fe^3+^ controlled “OFF–ON–OFF” fluorescent switch. By alternating the addition of HSO_4_^–^ and Fe^3+^, the switch could be reversibly performed for at least three cycles with little fluorescence efficiency loss (Fig. 5b). Moreover, **EuG** and **TbG** could selectively fluorescence “turn-on” sense OH^–^, while, upon the addition of H^+^ into the OH^–^-containing **EuG** or **TbG**, the fluorescence of **EuG** or **TbG** was quenched (Fig. 4 (line 16 and 17)), which was attributed to H^+^ competitively binding with OH^–^ and the Eu^3+^ or Tb^3+^ coordination with the gelator again. Meanwhile, **EuG** and **TbG** could act as H^+^ sensors.

**Fig. 6:**
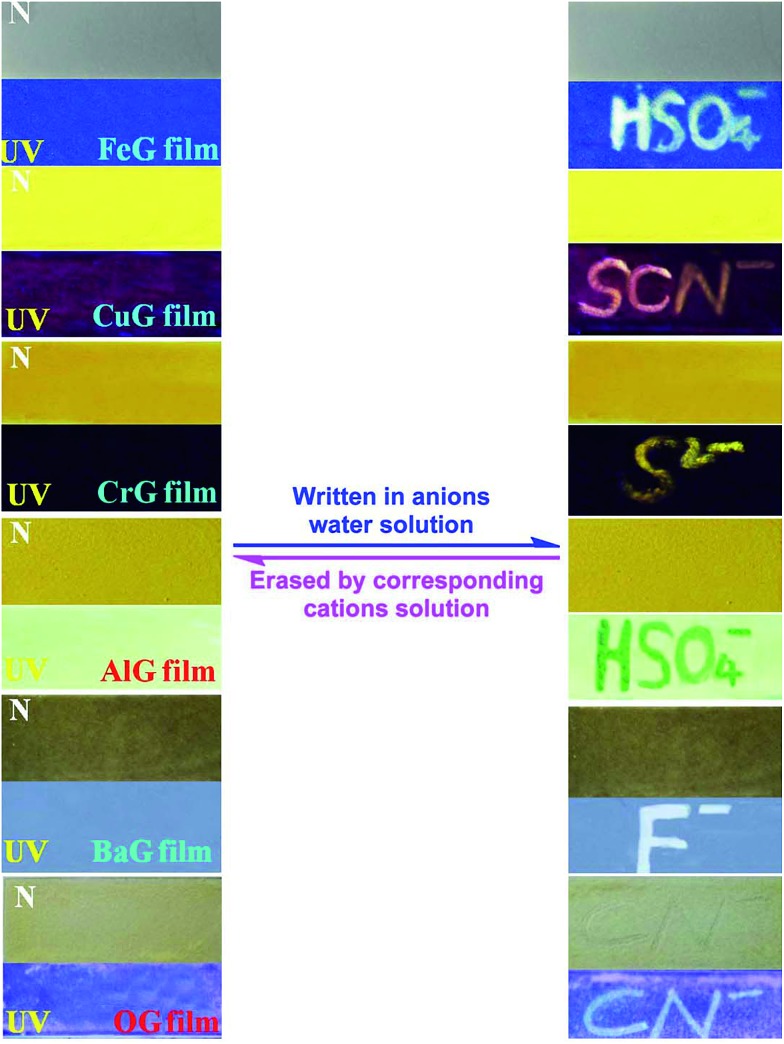
Writing and erasing of a natural light invisible image on supramolecular gel films. The photographs were taken at room temperature under natural light (N) and exposure to a 365 nm ultraviolet light (UV).

Moreover, these supramolecular gels could act as ion response fluorescent materials. For example, by pouring a heated solution of these gels onto a clean glass surface and drying in the air, we could obtain the corresponding ion response supramolecular films. As shown in Fig. 6, the **FeG** film has no fluorescence emission, when writing on the film with a writing brush dipped in HSO_4_^–^ water solution, a brilliant blue fluorescent writing appeared. This fluorescent image could be erased by brushing Fe^3+^ on the film again. Other supramolecular gels (Fig. 6) show similar properties also. Therefore, these supramolecular films could act as security display materials.

## Conclusions

In summary, by rationally introducing competitive binding interactions into a well designed supramolecular gel, a twenty-two member sensor array has been successfully developed. This sensor array could sense fourteen kinds of important ions such as CN^–^, F^–^, SCN^–^, Hg^2+^, Pb^2+^*etc.* with high selectivity and sensitivity in a water solution. This sensor array needed only one synthesized receptor. Moreover, using this method, we also obtained a series of ion response fluorescent supramolecular materials, which could act as erasable security display materials. Therefore, this is an efficient and simple way to develop a sensor array as well as stimuli–responsive supramolecular materials.

## Supplementary Material

Supplementary information
