# Supplementary material for: Rationally introduce multi-competitive binding interactions in supramolecular gels: a simple and efficient approach to develop multi-analyte sensor array
Source: Chem Sci. 2016 Apr 25;7(8):5341–6. doi: 10.1039/c6sc00955g (PMC6020755; doi:10.1039/c6sc00955g)
Supplement: Supplementary file 1 [file SC-007-C6SC00955G-s001.pdf]

Electronic Supplementary Material (ESI) for Chemical Science.
This journal is © The Royal Society of Chemistry 2016

Electronic Supporting Information

Rationally Introduce Multi-competitive Binding Interactions in Supramolecular Gels: A Simple and Efficient Approach to Develop Multi-analytes Sensor Array

Qi Lin, Tao-Tao Lu, Xin Zhu, Tai-Bao Wei, Hui Li, You-Ming Zhang**

Key Laboratory of Eco-Environment-Related Polymer Materials, Ministry of Education of China, Key Laboratory of Polymer Materials of Gansu Province, College of Chemistry and Chemical Engineering, Northwest Normal University, Lanzhou, Gansu, 730070, P. R. China.

Table of Contents

Materials and instruments

Scheme S1. The synthesis of **G**

Synthesis of gelator G

Figure S1. ^{1}H NMR Spectrum of **G**

Figure S2. ^{13}C NMR Spectrum of **G**

Figure S3. Mass Spectrum of **G**

Table S1. Gelation Property of Organogelator **G**

Figure S4. FT-IR spectra of (a) powder **G** and xerogel of organogel **OG**; (c) xerogel of **OG**, **OG**+Fe^{3+} and **OG+**Fe^{3+}+HSO_4^-; (b) xerogel of **OG**, and xerogel **OG** +**CN$^-$**.

Figure S5. Powder XRD patterns of powder and xerogel of **OG**.

Figure S6. SEM images of (a) **OG** xerogel; (b) **FeG** xerogel; (c) **FeG** xerogel treated with HSO_4^- in situ.

Figure S7. Fluorescence spectra of organogel of **OG** (in gelated state) in n-butyl alcohol (0.8% w/v).

Figure S8. Photographs of organogel of **OG** in n-butyl alcohol (0.8% w/v) and organogels of **OG** in the presence of various metal ions (Mg^{2+}, Ca^{2+}, Cr^{3+}, Fe^{3+}, Co^{2+}, Ni^{2+}, Cu^{2+}, Zn^{2+}, Ag^+, Cd^{2+}, Hg^{2+}, Pb^{2+}, Ba^{2+}, Sr^{2+}, Al^{3+}, La^{3+}, Y^{3+}, Ru^{3+}, Eu^{3+} and Tb^{3+}, **G**: metal ions =2 : 1) under (a) nature light (b) UV light.

Figure S9. Fluorescence spectra of organogel of **OG** (in gelated state) in n-butyl alcohol (0.8% w/v) and organogels of **OG** in the presence of various metal ions (using their perchloric salts chlorizated salts as the sources) added in 1 : 1 mol ratio with respect to **OG** (λ_{ex} = 300 nm).

Figure S10. Fluorescence spectra of supramoleculargel based sensor (in gelated state) (a)**CuG**, (b)**CrG**, (c)**BaG** and (d)**AlG** in the presence of various anions (F^-, Cl^-, Br^-, I^-, AcO^-, $H_2PO_4^-$, N_3^-, SCN^-, ClO_4^-, S^{2-} CN^- H^+ and OH^-); (e) **LaG**, (f) **EuG** and (g) **TbG** treated with OH^-; (h) **AlFeG** and (i) **AlCuG** treated with CN^-; (j) **AlCrG** treated with S^{2-}; (k) **AlHgG** treated with Cl^-; (l) **BaMgG** treated with I^-; (m) **BaCaG** treated with HSO_4^-; (n) **OG+CN$^-$**

treated with Fe^{3+}; (o) **OG** in the presence of various anions. (λ_{ex} = 300 nm).

Figure S11. Fluorescence spectra of supromaleculargel based sensors (in gelated state) with increasing concentration of guest ions: (a) **FeG** with HSO_4^- (b) **CuG** with SCN^-; (c) **CrG** with S^{2-}; (d) **BaG** with F^-; (e) **AlG** with HSO_4^-; (f) **AlFeG** with CN^-; (g) **AlHgG** with Cl^-; (h) **AlCrG** with S^{2-}; (i) **AlCuG** with CN^-; (j) **BaMgG** with I^-; (k) **BaCaG** with HSO_4^-; (l) **YG** with Pb^{2+}; (m) **RuG** with Al^{3+}; (n) **LaG** with Hg^{2+}; (o) **EuG** with Zn^{2+}; (p) **OG**+CN^- with Fe^{3+}; (q) **OG** CN^-. (λ_{ex} = 300 nm).

Figure S12. ^{1}H NMR spectra of (a) **G** (20 mg /ml^{-1}), (b) after addition of 0.5 equiv. of CN^- in $CDCl_3$ (0.4ml) and d_6-DMSO (0.1ml); (c) 1 equiv. of CN^-; (d) 2 equiv. of CN^{-1}.

Table S2. Detection limits of the supramolecular based sensor array for target ions.

Materials and instruments

All anions were used as the sodium salts while all cations were used as the perchlorate salts, which were purchased from Alfa Aesar and used as received. Fresh double distilled water was used throughout the experiment. Nuclear magnetic resonance (NMR) spectra were recorded on Varian Mercury 400 and Varian Inova 600 instruments. Mass spectra were recorded on a Bruker Esquire 6000 MS instrument. The X-ray diffraction analysis (XRD) was performed in a transmission mode with a Rigaku RINT2000 diffractometer equipped with graphite monochromated CuKa radiation (λ = 1.54073 Å). The morphologies and sizes of the xerogels were characterized using field emission scanning electron microscopy (FE-SEM, JSM-6701F) at an accelerating voltage of 8 kV. The infrared spectra were performed on a Digilab FTS-3000 Fourier transform-infrared spectrophotometer. Melting points were measured on an X-4 digital melting-point apparatus (uncorrected). Fluorescence spectra were recorded on a Shimadzu RF-5301PC spectrofluorophotometer.

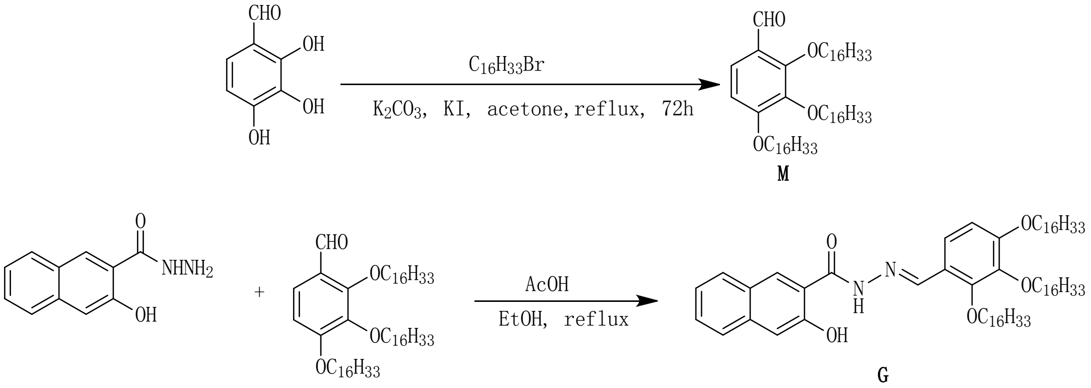

Scheme S1. The synthesis of **G**

Synthesis of gelator G

1. Synthesis of 2,3,4-tris-hexadecyloxy-benzaldehyde (**M**): 2,3,4-trihydroxy- benzaldehyde (5 mmol, 0.77g), 1-bromo-hexadecane (16 mmol, 4.88 g), K_2CO_3 (30 mmol, 4.14 g) and KI (2 mmol, 0.332 g) were added to 40 ml acetone. The reaction mixture was stirred under refluxing in nitrogen conditions for 36 hours. After removing the solvent, the precipitate was dissolved in $CHCl_3$ and then being washed by H_2O and saturated sodium chloride aqueous solution successively. The product 2,3,4-tris-hexadecyloxy-benzaldehyde (**M**) was obtained after evaporating the solvent. (yield: 90%). ^1H NMR ($CDCl_3$, 400 MHz): δ, 10.26 (s, 1H, -CH=O), 7.57 (d, 1H, J = 8.0 Hz, -ArH), 6.71 (d, 1H, J = 8.0 Hz, -ArH), 3.97-4.17 (m, 6H, -OCH_2), 1.74-1.87 (m, 6H, -CH_2), 1.26-1.31 (m, 78H, -CH_2), 0.88 (t, J = 8.0 Hz, 9H, -CH_3). MS-ESI calcd for $C_{55}H_{103}O_4$ [**G** + H]$^+$: 827.7800; found: 827.6445.

2. Synthesis of gelator G: 2,3,4-tris-hexadecyloxy-benzaldehyde (**M**) (1 mmol), 3-hydroxy-naphthohydrazide (1 mmol) and glacial acetic acid (0.05 mmol, as a catalyst) were added to ethanol (15 mL). Then the reaction mixture was stirred under refluxed conditions for 24 hours, after removing the solvent, yielding the precipitate of **G** (yield, 79%). ^1H NMR ($CDCl_3$, 400 MHz): δ, 11.22 (s, 1H,-OH) δ, 9.51 (s, 1H,-NH), 8. 51 (s, 1H, -N=CH), 8.10 (s, 1H, -ArH), 7.80-7.79 (d, 1H, J = 4.0 Hz, -ArH), 7.71-7.70 (d, J = 4.0 Hz, 1H, -ArH), 7.58-7.56 (d, J = 8.0 Hz, 1H, -ArH), 7.50-7.49 (d, J = 4.0 Hz, 1H, -ArH), 7.34-7.36 (t, J = 8.0 Hz, 1H, -ArH), 6.63-6.72 (m, 2H, -ArH), 4.18-3.97 (m, 6H, -OCH_2), 1.85-1.75 (m, 6H, -OCH_2CH_2), 1.35-1.26 (m, 78H, -CH_2), 0.89-0.87 (t, J = 8.0 Hz, 9H, -CH_3). ^{13}C-NMR ($CDCl_3$, 100 MHz) δ/ppm 153.0, 139.2, 128.6, 128.1, 126.9, 126.2, 124.4, 122.3, 108.51, 74.8, 73.7, 68.8, 31.9, 30.3, 29.7, 29.7, 29.7, 29.6, 29.5, 29.4, 29.4, 26.2, 26.1, 26.10, 26.1, 22.7, 14.1. IR (KBr, cm-1) v: 3202 (broad peak, O-H, N-H), 1649 (C=O), 1590 (C=N); MS-ESI calcd for $C_{66}H_{111}N_2O_5$ [**G** + H]$^+$: 1011.8488; found: 1011.6870.

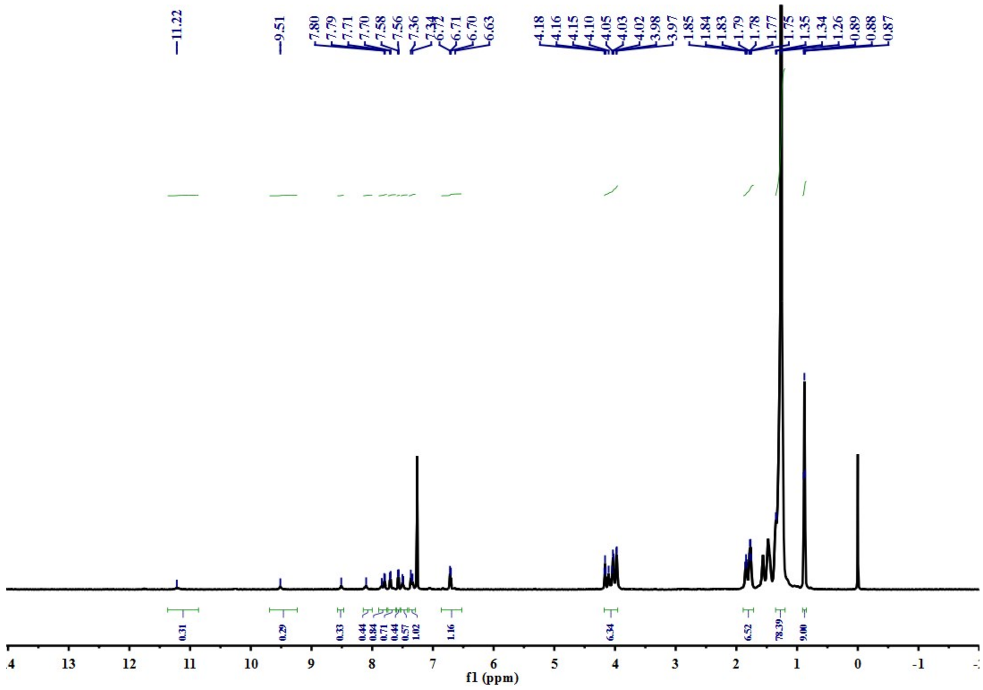

Figure S1. ^{1}H NMR Spectrum of **G**

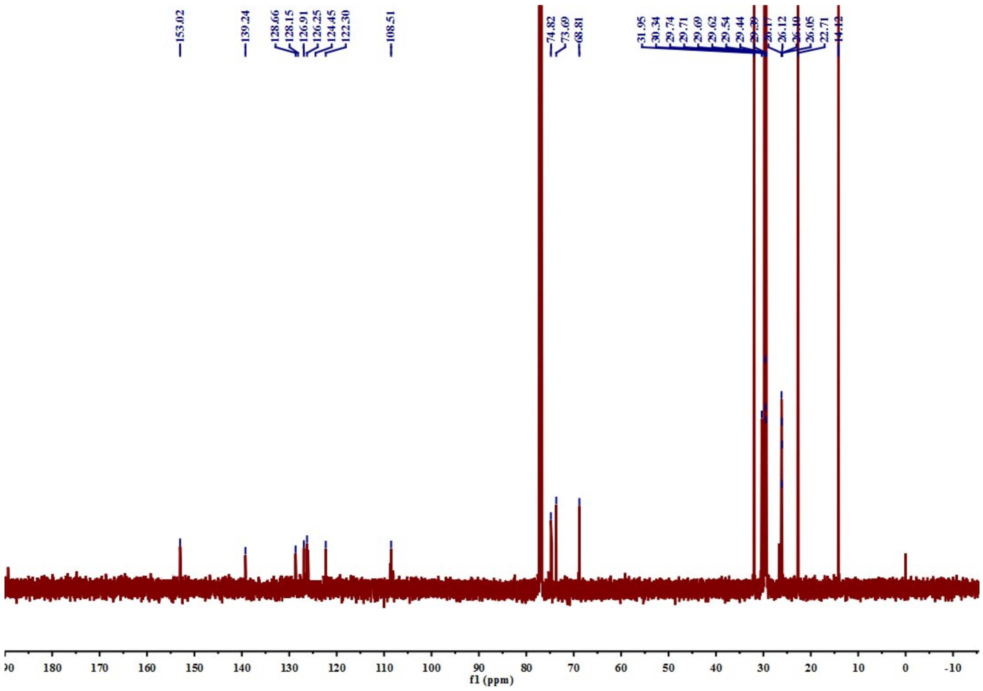

Figure S2. ^{13}C NMR Spectrum of **G**

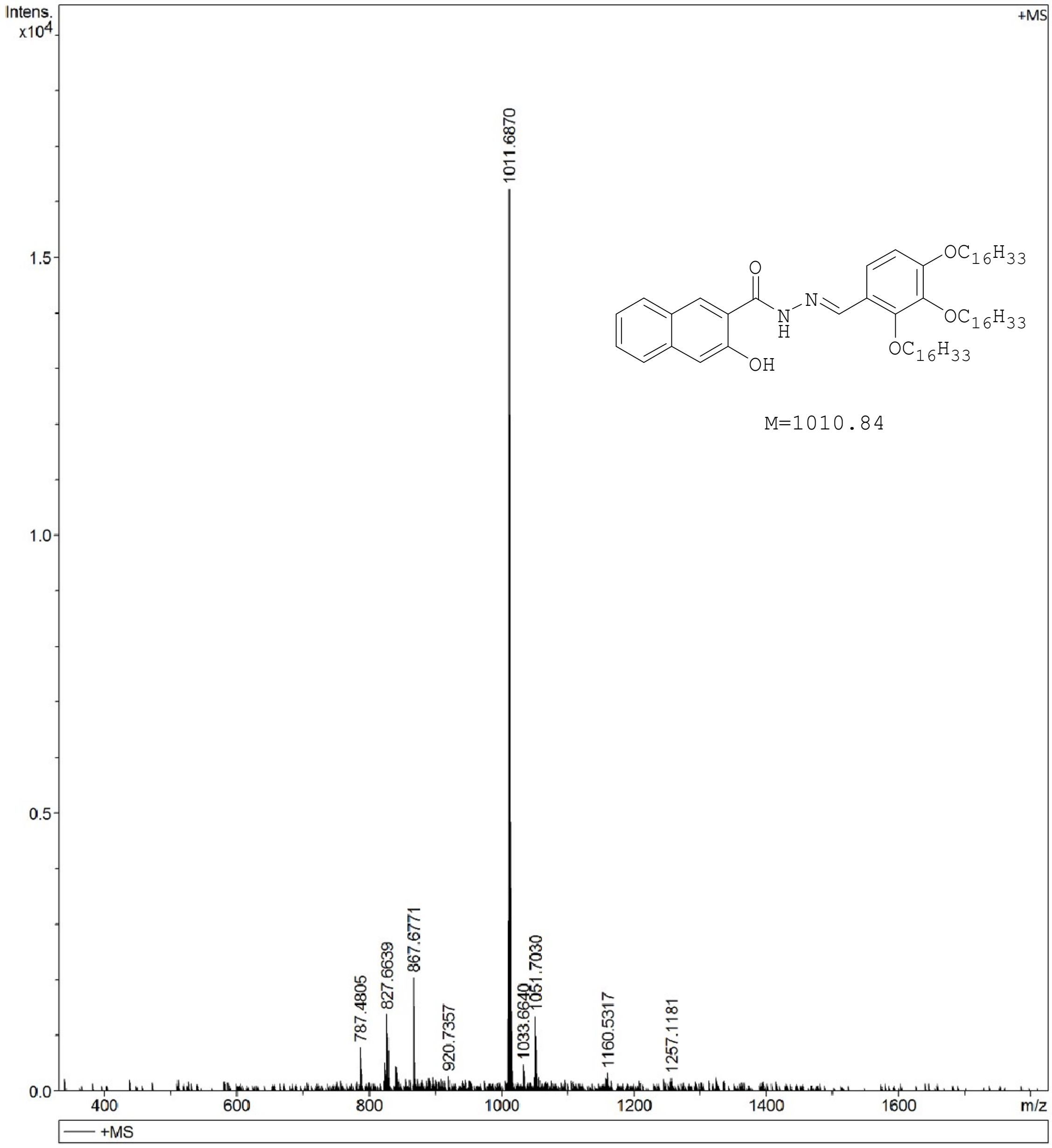

Figure S3. Mass Spectrum of **G**

Table S1. Gelation Property of Organogelator **G.**

Entry	Solvent	State[a]	CGC[b](%)	Tgel[c] (℃, wt%)
1	water	P	\	\
2	acetone	G	1.5	\
3	methanol	P	\	\
4	ethanol	G	2	54(2.5 %)
5	isopropanol	G	1	50(1.5 %)
6	isopentanol	G	0.8	49(1%)
7	acetonitrile	P	\	\
8	THF	S	\	\
9	DMF	G	1.5	49(2 %)
10	DMSO	G	4	48(4.5 %)
11	CCl_4	G	1	52(1.5 %)
12	n-hexane	G	1	51(1.5 %)
13	ethanediol	P	\	\
14	benzene	S	\	\
15	CH_2Cl_2	S	\	\
16	$CHCl_3$	S	\	\
17	CH_2ClCH_2Cl	S	\	\
18	petroleum ether	G	3	52(3.5%)
19	ethyl acetate	G	1	48(1.5%)
20	n-propanol	G	2	55(2.5%)
21	n-butyl alcohol	G	0.2	47(0.6%)
22	n-amyl alcohol	G	0.2	48(0.6%)
23	cyclohexanol	G	2	50(2.5%)
24	n-hexanol	G	1	49(1.5%)

[a]G, P and S denote gelation, precipitation and solution, respectively, c = 0.8%.

[b]The critical gelation concentration (wt%, 10mg/ml = 1.0%).

[c]The gelation temperature(℃).

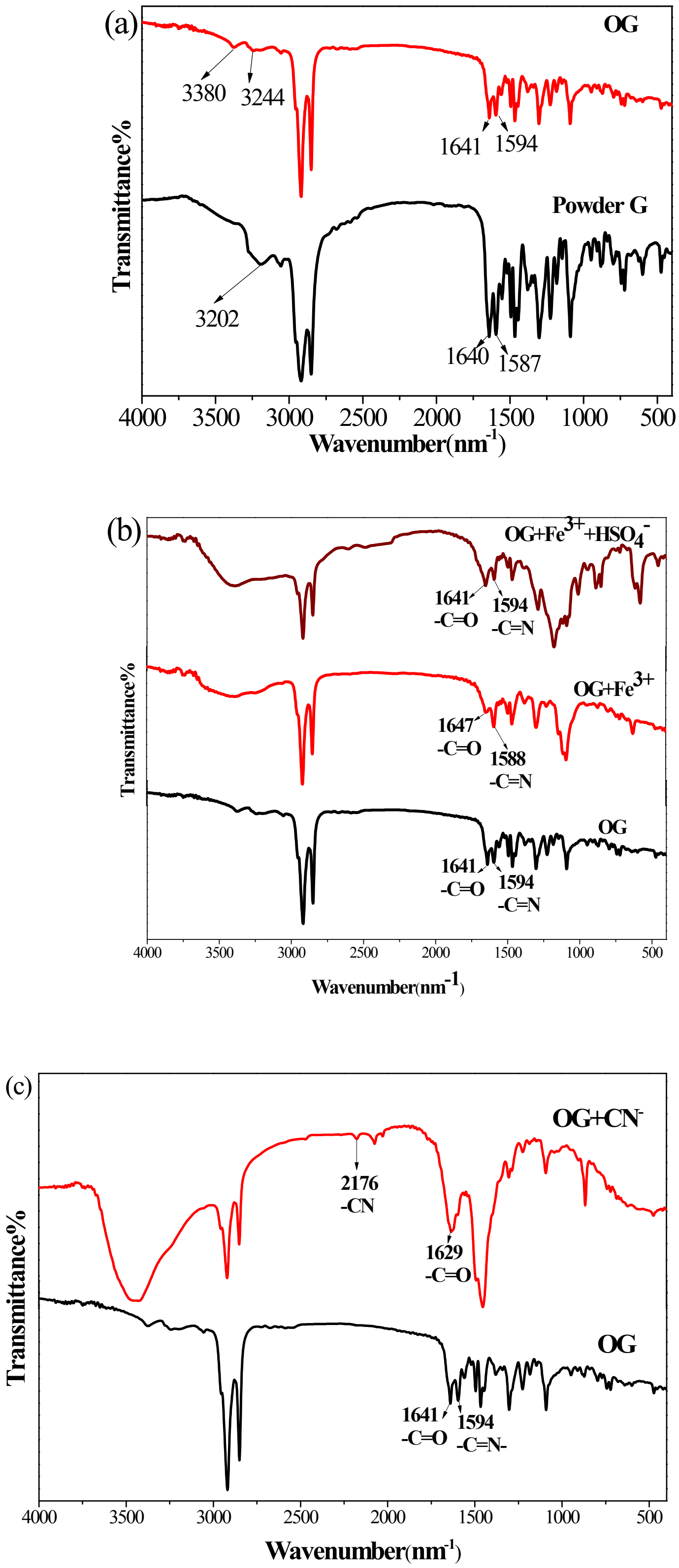

Figure S4. FT-IR spectra of (a) powder **G** and xerogel of organogel **OG**; (c) xerogel of **OG**, **OG**+Fe^{3+} and **OG**+Fe^{3+}+HSO_4^-; (b) xerogel of **OG**, and xerogel **OG** +**CN**$^-$.

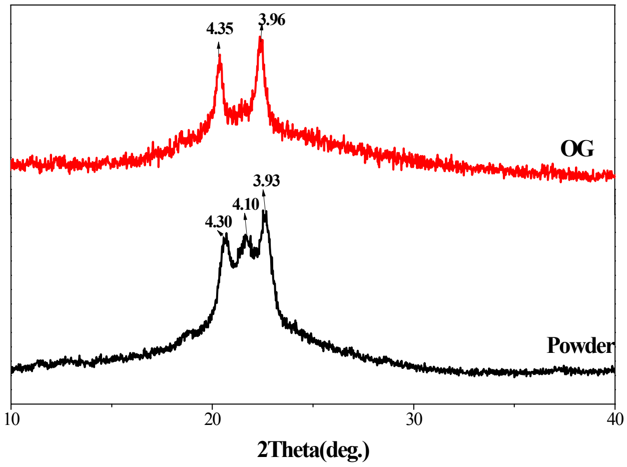

Figure S5. Powder XRD patterns of powder and xerogel of **OG**.

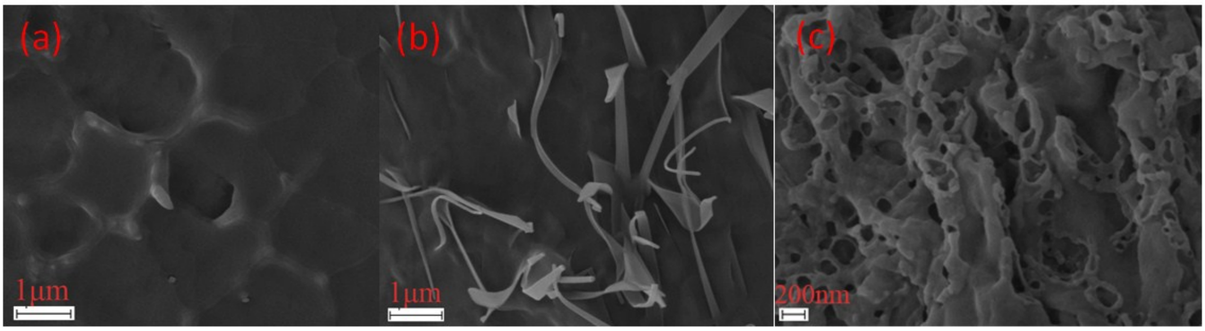

Figure S6. SEM images of (a) **OG** xerogel; (b) **FeG** xerogel; (c) **FeG** xerogel treated with HSO_4^- in situ.

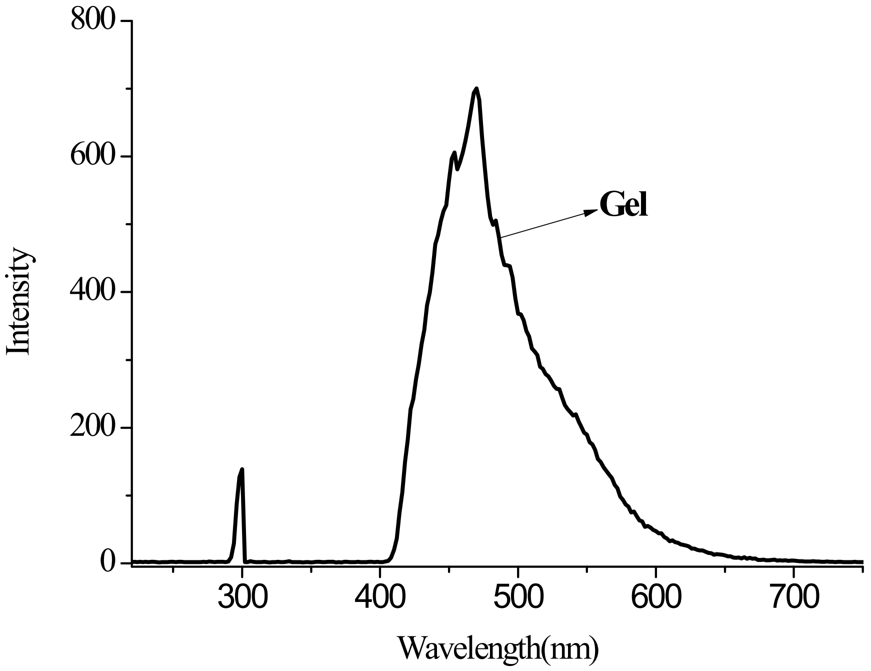

Figure S7 Fluorescence spectra of organogel of **OG** (in gelated state) in n-butyl alcohol (0.8% w/v).

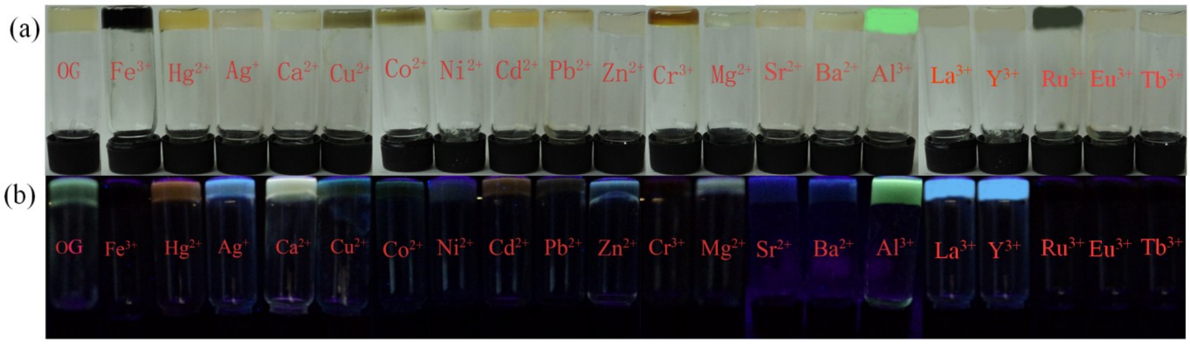

Figure S8. Photographs of organogel of **OG** in n-butyl alcohol (0.8% w/v) and organogels of **OG** in the presence of various metal ions (Mg^{2+}, Ca^{2+}, Cr^{3+}, Fe^{3+}, Co^{2+}, Ni^{2+}, Cu^{2+}, Zn^{2+}, Ag^{+}, Cd^{2+}, Hg^{2+}, Pb^{2+}, Ba^{2+}, Sr^{2+}, Al^{3+}, La^{3+}, Y^{3+}, Ru^{3+}, Eu^{3+} and Tb^{3+}, **G**: metal ions =2 : 1) under (a) nature light (b) UV light.

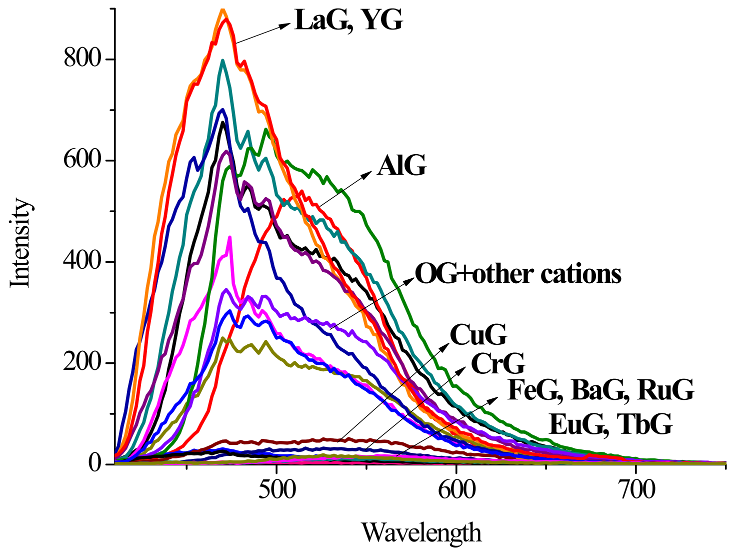

Figure S9. Fluorescence spectra of organogel of **OG** and **OG** (in gelated state) in the presence of various metal ions (Mg^{2+}, Ca^{2+}, Cr^{3+}, Fe^{3+}, Co^{2+}, Ni^{2+}, Cu^{2+}, Zn^{2+}, Ag^{+}, Cd^{2+}, Hg^{2+}, Pb^{2+}, Ba^{2+}, Sr^{2+}, Al^{3+}, La^{3+}, Y^{3+}, Ru^{3+}, Eu^{3+} and Tb^{3+}, **G**: metal ions =2 : 1) . (λ_{ex} = 300 nm).

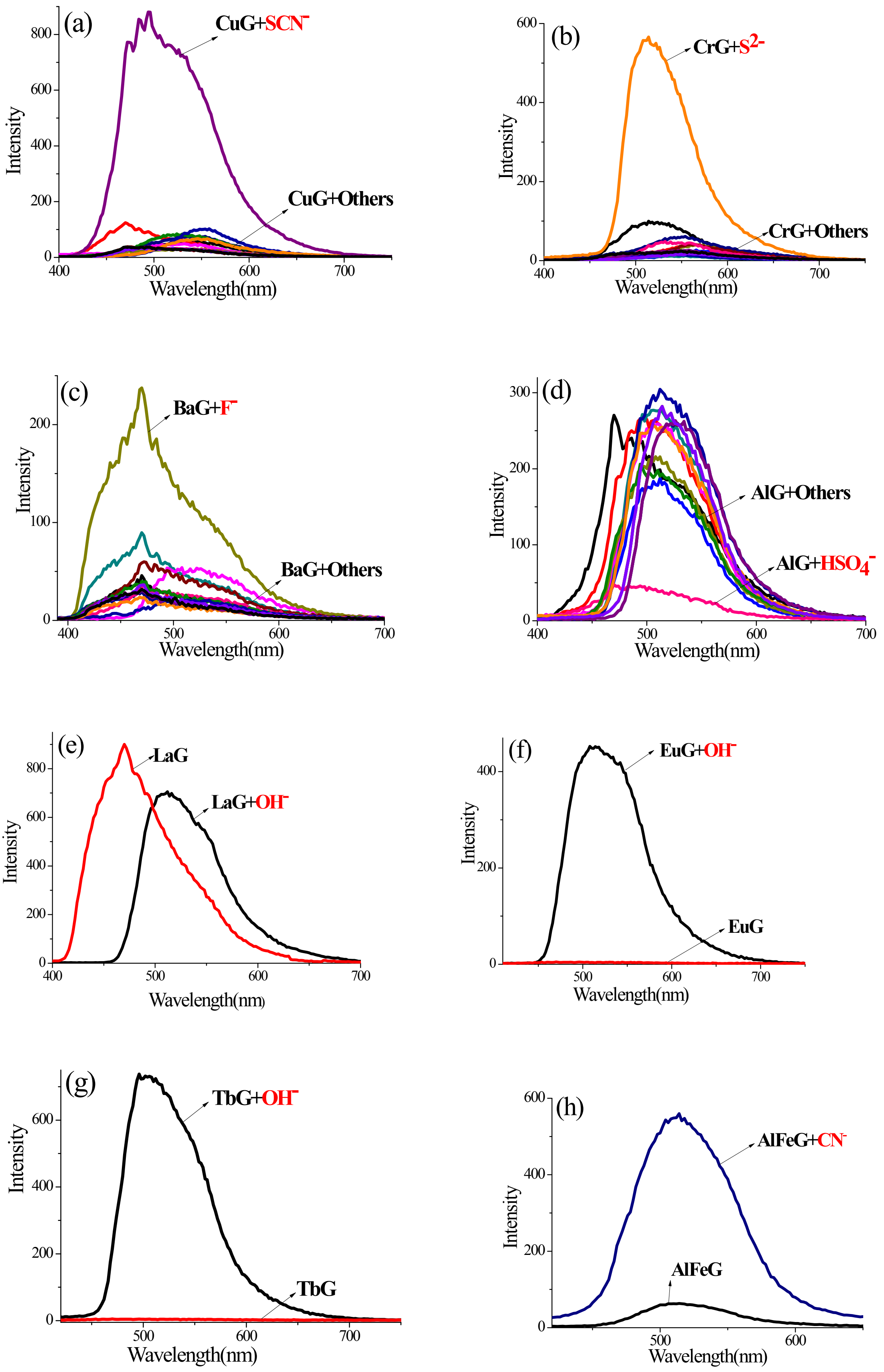
(a)
CuG+SCN-
CuG+Others
Intensity
Wavelength(nm)
(b)
CrG+S2-
CrG+Others
Intensity
Wavelength(nm)
(c)
BaG+F-
BaG+Others
Intensity
Wavelength(nm)
(d)
AlG+Others
AlG+HSO4-
Intensity
Wavelength(nm)
(e)
LaG
LaG+OH-
Intensity
Wavelength(nm)
(f)
EuG+OH-
EuG
Intensity
Wavelength(nm)
(g)
TbG+OH-
TbG
Intensity
Wavelength(nm)
(h)
AlFeG+CN-
AlFeG
Intensity
Wavelength(nm)

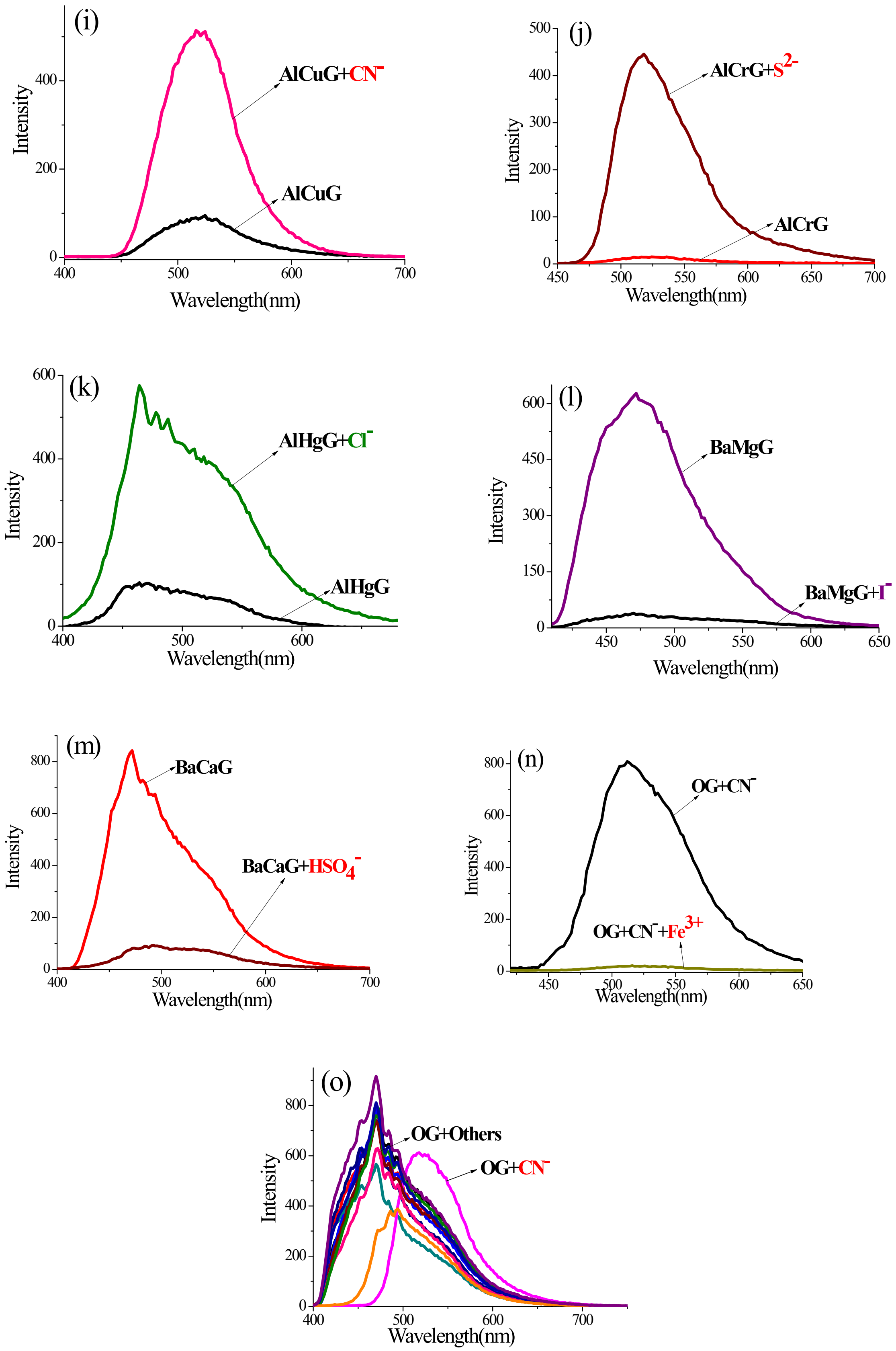

Figure S10. Fluorescence spectra of supramoleculargel based sensor (in gelated state) (a)**CuG**, (b)**CrG**, (c)**BaG** and (d)**AlG** in the presence of various anions (F^-, Cl^-, Br^-, I^-, AcO^-, $H_2PO_4^-$, N_3^-, SCN^-, ClO_4^-, S^{2-} CN^- H^+ and OH^-); (e) **LaG**, (f) **EuG** and (g) **TbG** treated with OH^-; (h) **AlFeG** and (i) **AlCuG** treated with CN^-; (j) **AlCrG** treated with S^{2-}; (k) **AlHgG** treated with Cl^-; (l) **BaMgG** treated with I^-; (m) **BaCaG** treated with HSO_4^-; (n) **OG+CN⁻** treated with Fe^{3+}; (o) **OG** in the presence of various anions. (λ_{ex} = 300 nm).

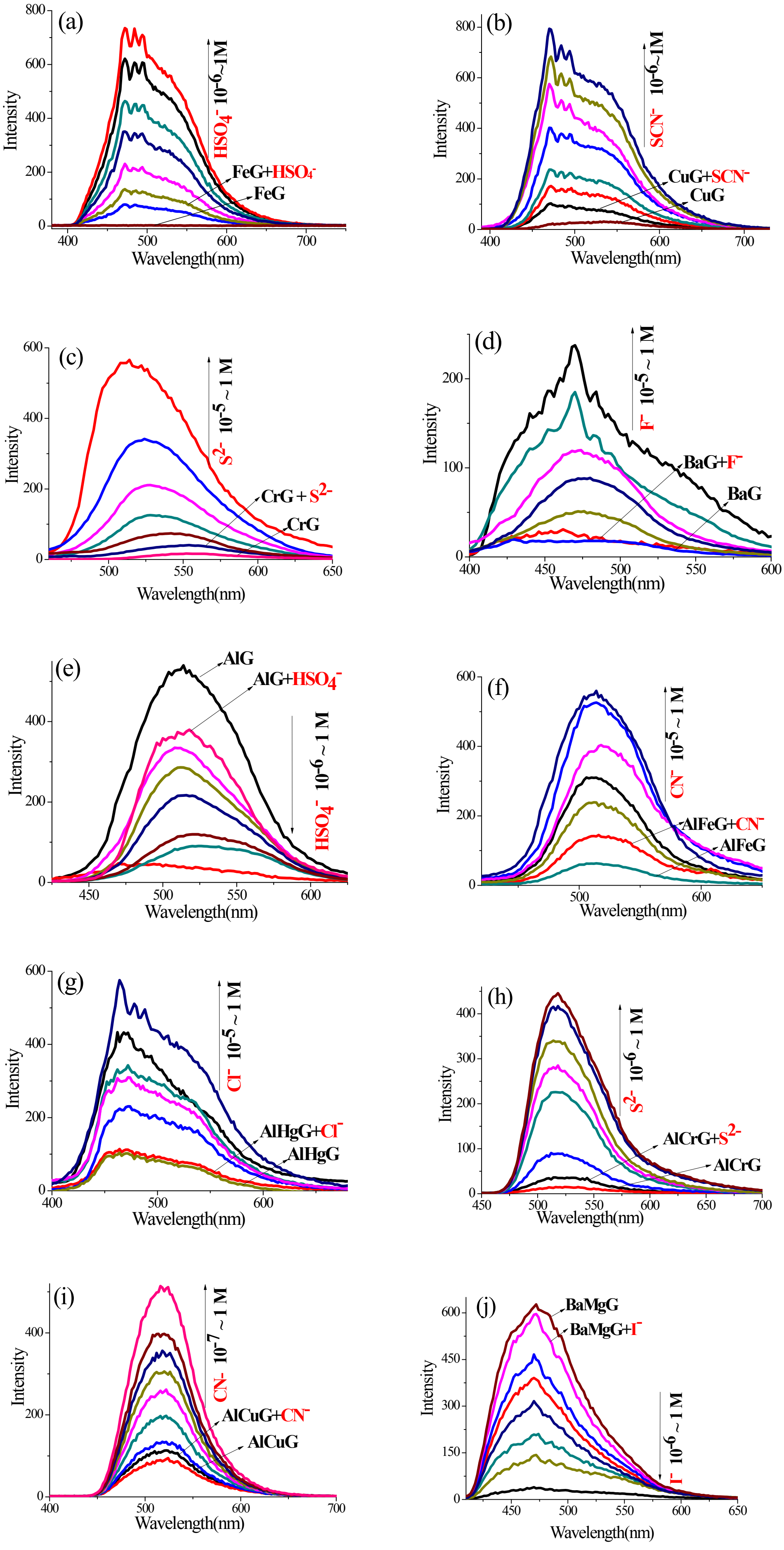

(a)
Intensity
Wavelength(nm)
HSO4- 10-6~1M
FeG+HSO4-
FeG
(b)
Intensity
Wavelength(nm)
SCN- 10-6~1M
CuG+SCN-
CuG
(c)
Intensity
Wavelength(nm)
S2- 10-5 ~ 1 M
CrG + S2-
CrG
(d)
Intensity
Wavelength(nm)
F- 10-5 ~ 1 M
BaG+F-
BaG
(e)
Intensity
Wavelength(nm)
AlG
AlG+HSO4-
HSO4- 10-6 ~ 1 M
(f)
Intensity
Wavelength(nm)
CN- 10-5 ~ 1 M
AlFeG+CN-
AlFeG
(g)
Intensity
Wavelength(nm)
Cl- 10-5 ~ 1 M
AlHgG+Cl-
AlHgG
(h)
Intensity
Wavelength(nm)
S2- 10-6 ~ 1 M
AlCrG+S2-
AlCrG
(i)
Intensity
Wavelength(nm)
CN- 10-7 ~ 1 M
AlCuG+CN-
AlCuG
(j)
Intensity
Wavelength(nm)
BaMgG
BaMgG+I-
I- 10-6 ~ 1 M

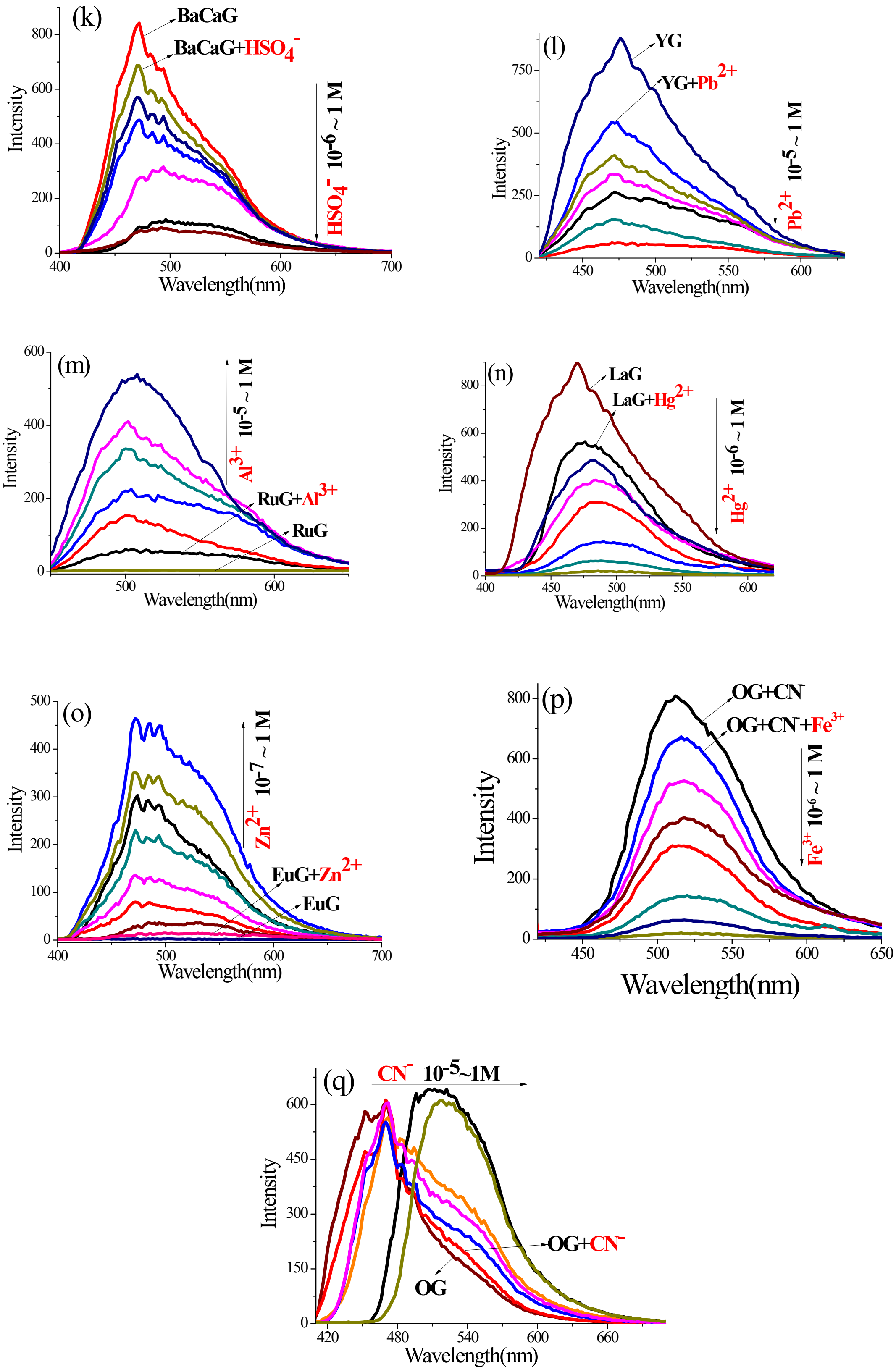

Figure S11. Fluorescence spectra of supromalecular gel based sensors (in gelated state) with increasing concentration of guest ions: (a) **FeG** with HSO_4^- (b) **CuG** with SCN^-; (c) **CrG** with S^{2-}; (d) **BaG** with F^-; (e) **AlG** with HSO_4^-; (f) **AlFeG** with CN^-; (g) **AlHgG** with Cl^-; (h) **AlCrG** with S^{2-}; (i) **AlCuG** with CN^-; (j) **BaMgG** with I^-; (k) **BaCaG** with HSO_4^-; (l) **YG** with Pb^{2+}; (m) **RuG** with Al^{3+}; (n) **LaG** with Hg^{2+}; (o) **EuG** with Zn^{2+}; (p) **OG**+CN^- with Fe^{3+}; (q) **OG** CN^-. (λ_{ex} = 300 nm).

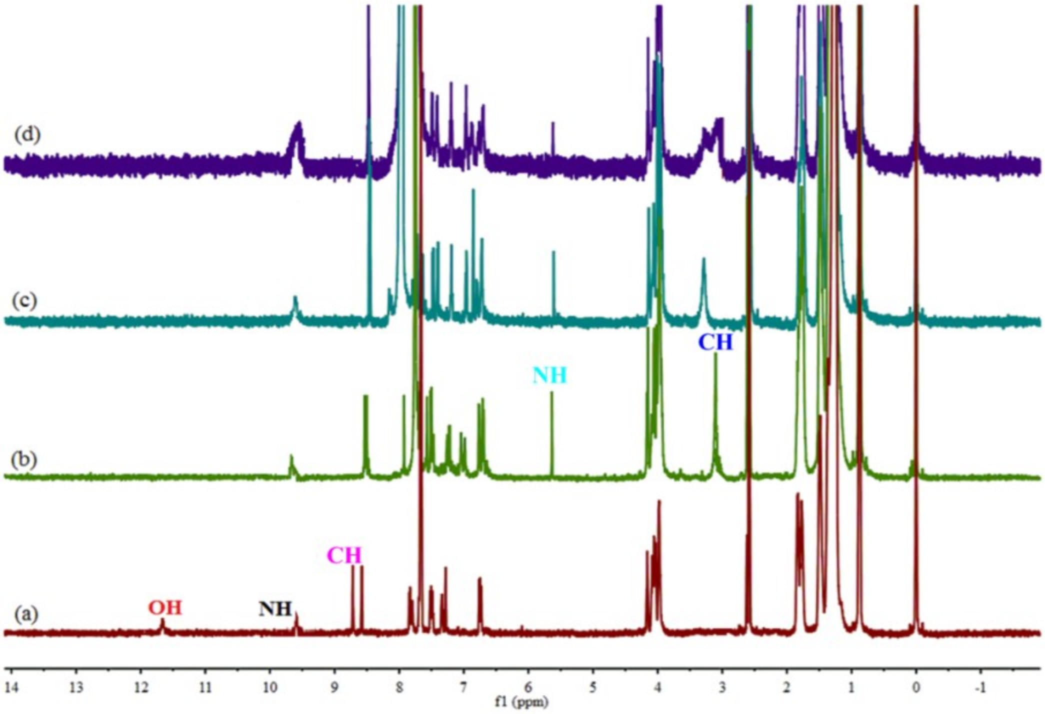

Figure S12 ^{1}H NMR spectra of (a) **G** (20 mg /ml^{-1}), (b) after addition of 0.5 equiv. of CN^- in $CDCl_3$ (0.4ml) and d_6-DMSO (0.1ml); (c) 1 equiv. of CN^-; (d) 2 equiv. of CN^{-1}.

Table S2. Detection limits of the supramolecular based sensor array for target ions.

sensor	ions	detection limits/M^{-1}
**OG**	CN^-	10^{-5}
**FeG**	HSO_4^-	10^{-6}
**CuG**	SCN^-	10^{-6}
**CrG**	S^{2-}	10^{-5}
**BaG**	F^-	10^{-5}
**AlG**	HSO_4^-	10^{-6}
**AlFeG**	CN^-	10^{-5}
**AlHgG**	Cl^-	10^{-5}
**AlCuG**	CN^-	10^{-7}
**AlCrG**	S^{2-}	10^{-6}
**BaMgG**	I^-	10^{-6}
**BaGaG**	HSO_4^-	10^{-6}
**OG+CN$^-$**	Fe^{3+}	10^{-6}
**LaG**	Hg^{2+}	10^{-6}
**YG**	Pb^{2+}	10^{-5}
**RuG**	Al^{3+}	10^{-5}
**EuG**	Zn^{2+}	10^{-7}
